# Autonomic dysfunction and risk of severe hypoglycemia among individuals with type 2 diabetes

**DOI:** 10.1172/jci.insight.156334

**Published:** 2022-11-22

**Authors:** Arnaud D. Kaze, Matthew F. Yuyun, Rexford S. Ahima, Michael R. Rickels, Justin B. Echouffo-Tcheugui

**Affiliations:** 1 Department of Medicine, University of Maryland Medical Center, Baltimore, Maryland, USA.; 2 Department of Medicine, LifePoint Health, Danville, Virginia, USA.; 3 Department of Medicine, Division of Cardiology, Veteran Affairs Boston Healthcare System/Harvard Medical School, Boston, Massachusetts, USA.; 4 Department of Medicine, Division of Endocrinology, Diabetes and Metabolism, Johns Hopkins School of Medicine, Baltimore, Maryland, USA.; 5 Division of Endocrinology, Diabetes and Metabolism, Hospital of the University of Pennsylvania, Philadelphia, Pennsylvania, USA.

**Keywords:** Cardiology, Endocrinology, Diabetes

## Abstract

There are limited data on the link between cardiac autonomic neuropathy (CAN) and severe hypoglycemia in type 2 diabetes. Here, we evaluated the associations of CAN with severe hypoglycemia among 7,421 adults with type 2 diabetes from the Action to Control Cardiovascular Risk in Diabetes study. CAN was defined using ECG-derived measures. Cox’s and Andersen-Gill regression models were used to generate HRs (HRs) for the first and recurrent severe hypoglycemic episodes, respectively. Over 4.7 years, there were 558 first and 811 recurrent hypoglycemic events. Participants with CAN had increased risks of a first episode or recurrent episodes of severe hypoglycemia. The intensity of glycemic management modified the CAN association with hypoglycemia. In the standard glycemic management group, compared with those of participants without CAN, HRs for a first severe hypoglycemia event and recurrent hypoglycemia were 1.58 and 1.96, respectively. In the intensive glycemic management group, HRs for a first severe hypoglycemia event and recurrent hypoglycemia were 1.10 and 1.24, respectively. In summary, CAN was independently associated with higher risks of a first hypoglycemia event and recurrent hypoglycemia among adults with type 2 diabetes, with the highest risk observed among those on standard glycemic management.

## Graphical abstract

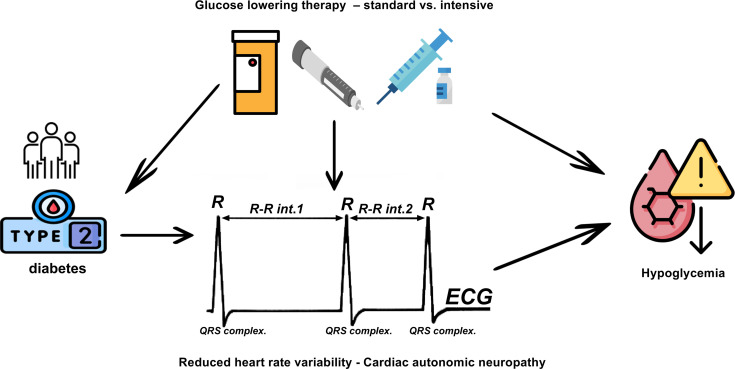

## Introduction

Type 2 diabetes mellitus is common in the United States (1). Hypoglycemia has been a key limiting factor in the glycemic management of individuals with type 1 and type 2 diabetes mellitus (2). Indeed, hypoglycemia is associated with poor quality of life (3), and it causes significant morbidity and mortality burdens in people with type 2 diabetes (4). Hypoglycemic unawareness, defined as hypoglycemia-associated autonomic failure (HAAF), is known to increases the risk of severe hypoglycemia (5). While hypoglycemic unawareness tends to co-occur with cardiac autonomic neuropathy (CAN), the two conditions are distinct and do not necessarily have any causal link (6, 7). There have been suggestions that autonomic dysfunction, including CAN, directly contributes to the development of impaired awareness of hypoglycemia (8). Autonomic dysfunction is a highly common complication of type 2 diabetes, with a prevalence as high as 34% (9). Data from experimental studies suggest a link between CAN and hypoglycemic episodes in type 1 diabetes mellitus (10, 11). However, large-scale human data on the links between autonomic dysfunction and severe hypoglycemia in people with type 2 diabetes are scarce. Furthermore, whether intensive glycemic control modifies the associations between autonomic dysfunction and severe hypoglycemic events among individuals with type 2 diabetes is unknown.

In the present study, using data from the Action to Control Cardiovascular Risk in Diabetes (ACCORD) trial, we investigated the relationship between CAN and the incidence of severe hypoglycemia (both the first episode of hypoglycemia and recurrent hypoglycemia). We also assessed whether these associations varied across the glycemic treatment arms of ACCORD.

## Results

### Baseline characteristics of study participants.

A total of 7,421 participants (mean age, 62.3 [S,D 6.5] years; 40.9% women and 59.1% men; 61.9% White, 18.8% Black, and 7.7% Hispanic; 11.6% of participants had other ancestry) were included in our investigation. Supplemental Table 1 (supplemental material available online with this article; https://doi.org/10.1172/jci.insight.156334DS1) displays the baseline characteristics of participants excluded compared with those included in the final sample; the reasons for exclusion are detailed in Supplemental Figure 1.

Among the included participants (Table 1 and Supplemental Table 2), the prevalence of CAN was 19.5% (*n* = 1,448). Participants with CAN were more frequently men, White (less frequently Black), current smokers, and insulin users. They also had higher BMI, resting heart rate, hemoglobin A_1C_ (HbA_1C_), total-to-HDL cholesterol ratio, longer duration of diabetes. Additionally, they were less likely to be on beta blockers and had a lower estimated glomerular filtration rate.

### Incidence of severe hypoglycemic events by CAN.

Over a median follow-up of 4.7 years (IQR, 3.9–5.6 years), there were 558 first severe hypoglycemia events (incidence rate [IR], 16.6/1,000 person-years, [95% CI, 15.3–18.1]) and 811 recurrent severe hypoglycemia events (IR, 24.5/1,000 person-years, [95% CI, 22.9–26.3]).

The incidence of severe hypoglycemia and recurrent severe hypoglycemia were highest among those with CAN undergoing intensive glycemic management; the IRs were 28.5 (95% CI, 23.1–35.0) and 50.5 (95% CI, 43.1–59.0). In multivariable analyses, CAN was associated with greater risk of severe hypoglycemia. The HR for the first episode of severe hypoglycemia among participants with CAN (vs. no CAN) was 1.23 (95% CI, 1.01–1.50); the equivalent HR for recurrent severe hypoglycemia was 1.46 (95% CI, 1.16–1.84) (model 4, Table 2).

**Figure 1:**
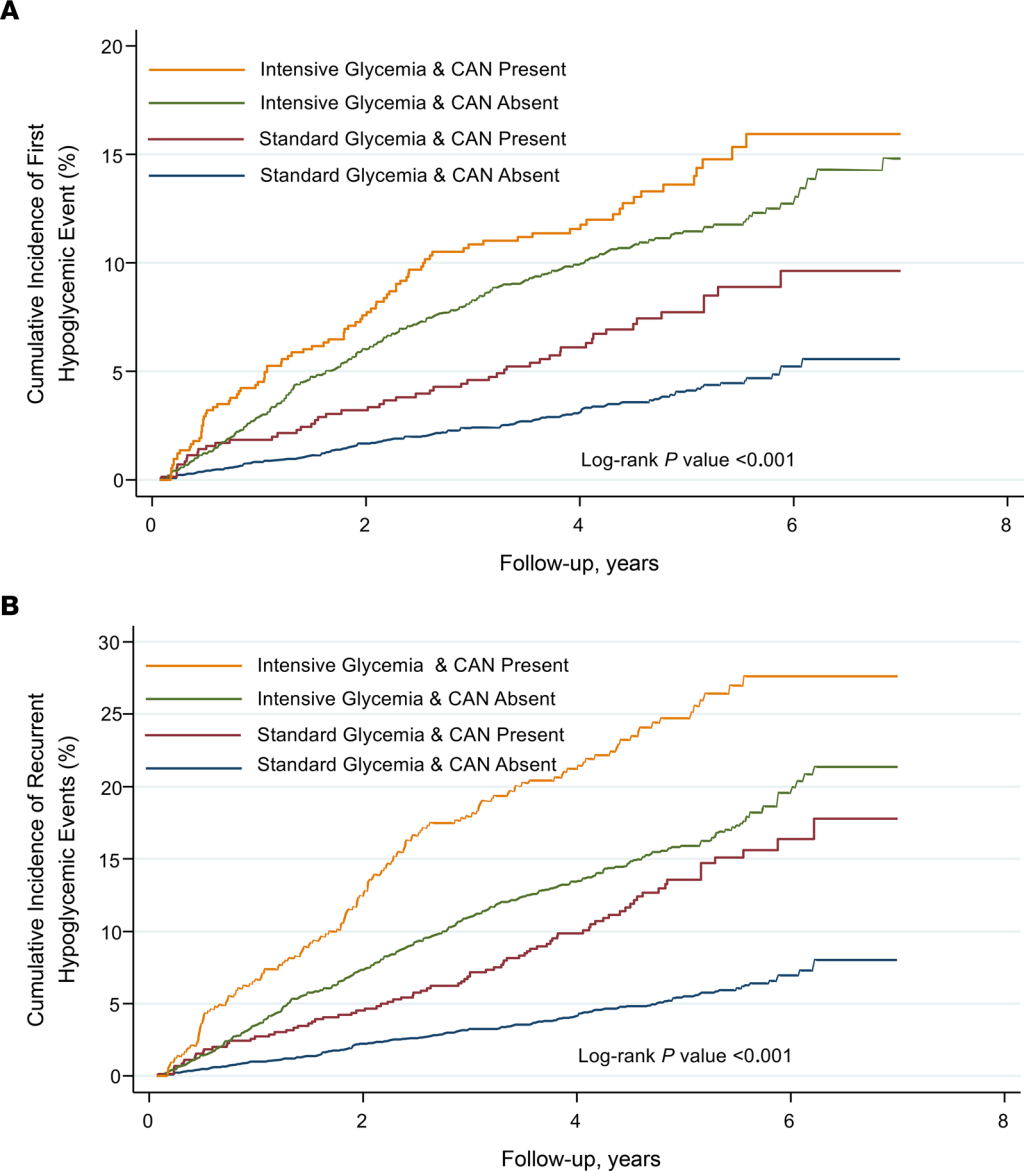
Cumulative incidence of hypoglycemic events. Cumulative incidence of first (**A**) and recurrent (**B**) severe hypoglycemic events by glycemia treatment arm and CAN. Severe hypoglycemia was defined as symptomatic, severe hypoglycemia requiring medical assistance. CAN, cardiac autonomic neuropathy.

In unadjusted analyses, the cumulative incidence of first and recurrent severe hypoglycemia was greater among participants in the intensive glycemic control arm with CAN and lower among those in the standard glycemic control arm without CAN (Figure 1, P < 0.001, log rank).

We found a significant interaction by glycemic treatment group (*P* for interaction = 0.004 for first episode of severe hypoglycemia; *P* for interaction = 0.026 for recurrent severe hypoglycemia) for the CAN and severe hypoglycemia associations. Consequently, we conducted analyses stratified by the intensity of the glycemic management strategy.

### Stratified analyses by the intensity of glycemic treatment.

In multivariable adjusted analyses, in the analyses within glucose-lowering treatment groups, there was a positive association between the presence of CAN and hypoglycemia (model 4, Table 3), which was more pronounced among individuals who received a standard glycemic management (HR, 1.58 [95% CI, 1.13–2.23] and 1.96 [95% CI, 1.33–2.90] for first hypoglycemic and recurrent hypoglycemic events, respectively). The corresponding estimates in the intensive glycemic management group showed less strong associations of CAN with hypoglycemia (HR, 1.10 [95% CI, 0.86–1.40] and 1.24 [95% CI, 0.93–1.65] for first hypoglycemic and recurrent hypoglycemic events, respectively).

### Analyses by cross categories of CAN and intensity of glycemic treatment.

In multivariable adjusted analyses, including cross categories of CAN and intensity of glycemic management, compared with participants in the standard glycemic control group without CAN, the HRs for a first episode of severe hypoglycemia were 1.68 (95% CI, 1.21–2.35) for those in standard glycemic control group with CAN, 3.15 (95% CI, 2.50–3.98) for those in the intensive glycemic control arm without CAN, and 3.37 (95% CI, 2.50–4.55) for those in the intensive glycemic control arm with CAN. The corresponding HRs for recurrent severe hypoglycemia were 2.09 (95% CI, 1.42–3.08), 3.30 (95% CI, 2.56–4.26), and 4.03 (95% CI, 2.85–5.70), respectively (model 4, Table 4).

We did not find any significant statistical interaction by age, sex, race, or use of insulin/ sulfonylurea.

### Supplemental analyses.

We repeated the analyses using a second definition of severe hypoglycemia as a symptomatic, severe hypoglycemic event requiring any assistance (either medical care or assistance from another individual). CAN remained positively associated with increased risks of a first severe hypoglycemic event (HR, 1.14 [95% CI, 0.97–1.34]) and recurrent hypoglycemic events (HR, 1.42 [95% CI, 1.12–1.80]), as detailed in Supplemental Table 3.

In the analyses stratified by glycemic treatment arms, the HRs for first hypoglycemic and recurrent hypoglycemic events in the standard glycemic management group were 1.43 (95% CI, 1.07–1.92) and 1.77 (95% CI, 1.25–2.50), respectively. The corresponding estimates in the intensive glycemic management group were 1.03 (95% CI, 0.84–1.25) and 1.30 (95% CI, 0.95–1.77), respectively (model 4, Supplemental Table 4).

Moreover, in the cross categories of CAN and treatment arm categories, compared with participants in the standard glycemic arm without CAN, the HRs for a first severe hypoglycemic event were 1.54 (95% CI, 1.16–2.04), 3.50 (95% CI, 2.90–4.23), and 3.52 (95% CI, 2.76–4.49) for those in the standard arm with CAN, the intensive arm without CAN, and the intensive arm with CAN, respectively. The equivalent HRs for recurrent hypoglycemic events were 1.83 (95% CI, 1.30–2.59), 3.89 (95% CI, 3.06–4.94), and 4.98 (95% CI, 3.48–7.13), respectively (Supplemental Table 5).

In analyses stratified by the use of beta blockers, the association of CAN (vs. no CAN) and the risks of first and recurrent severe hypoglycemia events were highest among participants in the standard treatment group on beta blockers (Supplemental Tables 6 and 7).

Among individuals with repeated data on heart rate variability (HRV), our assessment of the effect of change in CAN status from baseline to the 48-month visit showed that individuals whose status changed over time, moving from no CAN to CAN, tended to have a higher risk of hypoglycemia in the standard treatment group; the estimates of association were not significant (Supplemental Table 8).

## Discussion

We evaluated the associations of CAN with first and recurrent episodes of severe hypoglycemia in a large cohort of adults with type 2 diabetes who were enrolled in an intensive glucose-lowering trial: the ACCORD study. We observed that CAN was independently associated with greater risks of both first and recurrent episodes of severe hypoglycemia. In analyses stratified by the intensity glycemic treatment arm, the relative risks of first and recurrent episodes of severe hypoglycemia associated with CAN (as compared with no CAN) were greatest among those on standard glycemic management. We also found that the absolute risks of first and recurring hypoglycemic events was highest among those with CAN and undergoing intensive glycemic management.

We believe that our study is unique in that it investigates the association between CAN and incident hypoglycemic events among individuals with type 2 diabetes and assesses the influence of the extent of the intensity of glycemic control on this association. Prior investigations evaluating the adverse effects of autonomic dysfunction in people with type 2 diabetes focused predominantly on CVD and mortality as the outcomes, and they also reported an increased risk of adverse outcomes associated with CAN in this population (12–14). A prior prospective epidemiological study examined the association of CAN with severe hypoglycemia in type 2 diabetes (15) and showed results that are similar to our findings. However, this prior study was relatively smaller in size, lacked a racially diverse sample, and it did not assess the role of intensive glucose lowering on this association (15). Other studies of autonomic dysfunction and severe hypoglycemia have traditionally focused on individuals with type 1 diabetes (10, 11, 16, 17). In a small experimental study, individuals with type 1 diabetes (*n* = 44), 2 indices of glycemic variability reflective of hypoglycemic stress (low blood glucose index and area under the curve for hypoglycemia) were also associated with reduced HRV independent of the degree of glycemic control (10). Another study found that antecedent hypoglycemia was associated with the attenuation of vagal baroreflex sensitivity and the sympathetic response to hypotensive stress in 20 individuals with diabetes (18).

The relation between autonomic dysfunction and hypoglycemia in patients with diabetes mellitus is complex, incompletely understood, and of unclear directionality. Several mechanisms have been suggested to explain the vicious cycle that leads to recurrent hypoglycemic episodes in individuals with diabetes mellitus. One such pathway is that of HAAF, which is characterized by compromised defenses against hypoglycemia (2, 18, 19). Indeed, in HAAF, recent antecedent iatrogenic hypoglycemia causes defective glucose counterregulation (reduced epinephrine response to falling glucose levels in the setting of an absent glucagon response) and hypoglycemia unawareness (decreased autonomic and related neurogenic symptom responses), thus creating and perpetuating a vicious cycle of recurrent hypoglycemia (2, 18, 19). It is possible that among individuals with preexisting autonomic dysfunction (CAN), HAAF may be a frequent and preeminent phenomenon, more so than among those without a preexisting CAN (11, 17, 20), which is corroborated by a higher frequency of recurrent severe hypoglycemic events among those with CAN observed on our study. Furthermore, there is some experimental evidence from animal models and humans (mainly those with type 1 diabetes) that autonomic dysfunction contributes to impaired counterregulatory responses to insulin-induced hypoglycemia and to HAAF (17, 21).

Our cross categories of CAN and intensity of glycemic control categories showed that the absolute risk of severe hypoglycemic events is highest among individuals with CAN undergoing intensive glucose lowering. This is consistent with prior documented evidence of defective glucose counterregulation after strict glycemic control (22, 23). Indeed, strict glycemic control can lead to a reduction of the glucose threshold required to stimulate the release of epinephrine, growth hormone, cortisol, and consequently hepatic glucose output and generation of autonomic symptoms (22, 23), likely operating through central adaptation to frequent exposure to nonsevere hypoglycemia (20). It is possible that such alterations may have contributed to the observed greater incidence of severe hypoglycemic events in the intensive glycemic group in our study. There is also a possibility that antecedent hypoglycemia (prior to the entry in the ACCORD study) could have led to an impairment in cardiac autonomic function, with reduced baroreflex sensitivity and sympathetic response to a hypotensive stress, which will then contribute to the perpetuation of the vicious circle of hypoglycemia (18, 24).

Our findings should be interpreted in the context of a few limitations. First, CAN was defined using ECG-derived time-domain indices. We did not perform dynamic cardiovascular autonomic reflex tests (25), which represent the gold standard for diagnosis of CAN. Therefore, we may have missed some cases of CAN, thereby underestimating the association between CAN and severe hypoglycemia in our study population. Cardiovascular autonomic reflex tests may be difficult to implement on a large scale; thus, major guidelines recommend using HRV time-domain measures to define CAN in large epidemiological studies (26). Second, continuous glucose monitoring was not used for hypoglycemia detection; hence, it is possible that our study did not capture certain hypoglycemic episodes, for example, those occurring during sleep or in the setting of hypoglycemia unawareness (27). This could have led to misclassification of the outcome, resulting in a bias toward the null, and, thus, an underestimation of the magnitude of the association between CAN and hypoglycemia. Fourth, we did not have data on the dose of insulin used, which can influence the occurrence of hypoglycemia. However, we tried to capture the potential effect by accounting for the overall use of insulin and the intensity of the glucose-lowering regimen. Some of the subgroups or subset analyses, including, for example, the analyses on the progression of the CAN status, may have lacked power to detect differences in the hypoglycemia outcome. Fifth, the generalizability of our findings to the entire population of patients with type 2 diabetes is somewhat limited by the intrinsic characteristics of participants included in the ACCORD study, especially as a number of participants without EKG data were excluded from our analyses. Finally, our study used an observational design; hence, there is a possibility of unmeasured, residual confounding.

Despite these limitations, our study has several strengths. First, we analyzed a large cohort of individuals with type 2 diabetes. Second, we accounted for the intensity of glycemic management in assessing the association between CAN and incident hypoglycemia. Third, we performed a standardized ascertainment of severe hypoglycemic events, used several definitions of severe hypoglycemia, and conducted a rigorous adjustment for relevant confounders, including the use of insulin or insulin secretagogues, duration of diabetes, the degree and intensity of blood glucose control, estimated glomerular filtration rate, the use of beta blockers, and comorbidities.

The implications of this study are manifold for individuals with type 2 diabetes. Hypoglycemia remains a key limiting factor for glycemic control (2), which is sometimes compounded by hypoglycemic unawareness (28, 29). Our study complements prior experimental data by providing epidemiological evidence supporting the role of CAN in the occurrence of severe hypoglycemic episodes in people with type 2 diabetes. Our study showed that CAN is much more of a risk factor for hypoglycemia, particularly in the context of a nonintensive glucose-lowering therapy and much less so in the context of intensive therapy. It is possible that, in the context of intensive therapy, the latter supersedes CAN to influence hypoglycemia incidence. Our study suggests that a formal testing of the hypothesis that screening for CAN among individuals with diabetes would help prevent hypoglycemia should be conducted. The presence of autonomic dysfunction among individuals with type 2 diabetes could be used as an eligibility criterion for the use of a continuous glucose monitor, which may help prevent severe hypoglycemia. Indeed, the use of a continuous glucose-monitoring devices among individuals with type 1 diabetes with hypoglycemia unawareness significantly reduced the frequency of hypoglycemic episodes (30).

Overall, our study points to the importance of early CAN detection and the implementation of strategies to minimize the risk of hypoglycemia in individuals with type 2 diabetes. These strategies include discussing hypoglycemia at each visit and educating patients, encouraging frequent monitoring of blood glucose, using flexible therapeutic regimens with individualized glycemic goals accounting for conventional hypoglycemia risk factors (insulin doses, use of insulin secretagogues, missed meals, overnight fast, exercise, and alcohol ingestion). Further research is needed to investigate the potentials pathways relating CAN to severe hypoglycemia in this high-risk population.

In conclusion, in a large sample of adults with type 2 diabetes, CAN was independently associated with increased risks of first and recurrent severe hypoglycemic events. Moreover, the relative risk of hypoglycemia associated with CAN was higher among those undergoing standard glucose-lowering therapy, whereas the absolute risk of hypoglycemia was highest among participants in the intensive glycemia arm with cardiac autonomic dysfunction.

## Methods

### Study design.

We performed a secondary analysis of the data from the ACCORD study, the details of which have previously been reported (31). Briefly, the ACCORD study was a double 2-by-2 factorial clinical trial in which a total of 10,251 individuals with type 2 diabetes were recruited at 77 sites across the United States and Canada. The participants were enrolled in 2 noncontiguous periods (the first from January 2001 to June 2001, the second from January 2003 to October 2005) and were randomly assigned to receive either an intensive glucose-lowering intervention (intensive glycemic arm) with a glycated hemoglobin (HbA_1C_) goal of less than 6% or standard treatment aiming for an HbA_1C_ of 7.0%–7.9% (standard glycemic arm). Participants included in the ACCORD study were aged 40–79 years (with a history of CVD) or 55–79 years (with significant albuminuria, atherosclerosis, left ventricular hypertrophy, or a minimum of 2 CVD risk factors) (31).

For the present study, participants were excluded if they had an artificial pacemaker (*n* = 65), atrioventricular conduction defect (*n* = 445), atrial fibrillation/flutter (*n* = 125), premature beats and other arrhythmias (*n* = 814), and no ECG at baseline (*n* = 991) or a poor quality ECG (*n* = 390). After these exclusions, 7,421 participants were left for our analyses (Supplemental Figure 1). A subset of ACCORD participants had CAN assessed at baseline and at the 48-month visit (*n* = 4,111); they were therefore included in a subgroup analysis that assessed change in CAN status in relation to hypoglycemia.

### Autonomic dysfunction.

We defined autonomic dysfunction based on the presence of CAN, which was defined at baseline using HRV measures derived from 12-lead digitalized ECG recorded over 10 seconds, with the patient lying supine, after an overnight fast (GE MAC 1200 electrocardiograph system) (14). The ECG recordings were transmitted electronically to the reading center where they were analyzed and reviewed for their technical quality. Two time-domain indices of HRV were derived: SD of all normal-to-normal R-R intervals (SDNN) and root mean square of successive differences between normal-to-normal R-R intervals (rMSSD) (14). Lower HRV is an early marker of CAN in the course of diabetes mellitus (32). We defined CAN as both SDNN and rMSSD being below the fifth percentile of the general population distribution (SDNN < 8.2 ms and rMSSD < 8.0 ms) (33, 34).

### Severe hypoglycemia.

The ascertainment of severe hypoglycemia episodes was conducted at follow-up visits. Follow-up visit schedules were months 1, 2, 3, 4, and every 2 months thereafter for participants in the intensive glycemic management arm as well as those in the standard glycemia management plus intensive BP groups. For other participants in the standard glycemic management arm, follow-up visit schedules were months 1, 4, and every 4 months thereafter (31). At each visit, participants were queried as to whether they had experienced any episode of severe hypoglycemia.

For our main analysis, severe hypoglycemia was defined as symptomatic low glycemia, with either a blood glucose concentration of less than 50 mg/dL or symptoms that promptly resolved with oral carbohydrate, intravenous glucose, or subcutaneous or intramuscular glucagon and required medical assistance (defined as care at a hospital, an emergency room, or from medical personnel) (35, 36).

We analyzed both the first occurrence of an episode of severe hypoglycemia and recurrent episodes of severe hypoglycemia (all hypoglycemic events). We also considered a second definition of an episode of severe hypoglycemic, as a symptomatic, severe hypoglycemic event requiring any assistance (either medical care or assistance from another individual) (35, 36).

### Covariates.

We selected the covariates a priori based on their role as potential confounders. These included age, sex, race, treatment arm, cigarette smoking, alcohol consumption, BMI, HbA_1C_, duration of diabetes, use of insulin or sulfonylurea (31), baseline BP, use of BP-lowering medication (including beta blockers and calcium channel blockers), lipid variables, estimated glomerular filtration rate, history of prevalent CVD (defined as prior myocardial infarction; angina; stroke; history of coronary revascularization, history of carotid, history of peripheral revascularization; or history of heart failure) (31), and history of liver disease (as assessed by alanine transaminase levels).

### Statistics.

We compared the baseline characteristics of participants by groups defined using the glycemic management arm and CAN status. The comparisons were done using the 1-way ANOVA or Kruskal-Wallis test for continuous variables and the χ^2^ test for categorical variables. We assessed the time-to-event distributions for severe hypoglycemia by CAN status using the Kaplan-Meier curve and compared these distributions using the log-rank test. We calculated the IRs as the ratio of the cumulative number of severe hypoglycemic events to the total follow-up time. For time to first episode of severe hypoglycemia events, we used multivariable Cox proportional hazards regression models to compute HRs and associated 95% CIs relating CAN to incident first episode of severe hypoglycemia. For the recurrent severe hypoglycemia events, we used the Andersen-Gill regression model with robust variance estimation in order to account for the correlation between failure times within the same individual (37). We conducted 2 series of analyses: (a) a first set of analyses stratified by treatment groups, comparing those with CAN with those without CAN (reference group) within each glucose-lowering treatment group and (b) a second set of analyses with cross CAN and treatment arm comparisons, whereby persons without CAN and in the standard management group served as the reference group for all the other groups (persons with CAN in the standard management groups, persons without CAN in the intensive management group, and persons with CAN in the intensive management group).

All the regression models were built in a sequential manner as follows: model 1 included age, sex, race, and treatment arm; model 2 adjusted for variables in model 1 plus cigarette smoking, alcohol intake, BMI, systolic BP, use of beta blocker, use of calcium channel blocker, estimated glomerular filtration rate, duration of diabetes, HbA_1C_, and use of insulin/sulfonylurea; model 3 included model 2 with further adjustment for history of CVD at baseline; and model 4 included model 3 with further adjustment for alanine transaminase to capture liver function. We tested for statistical interaction by age, sex, race, and treatment arm.

In supplemental analyses, we conducted subgroup analyses among participants not on beta blockers compared with those on beta blockers to assess the effects of these medications on the estimates of association between CAN and incident hypoglycemia. In the subset of participants with repeated HRV measures, we assessed whether the change in CAN status over time (from baseline to 48 months) was associated with the incidence of hypoglycemia. We limited this analyses of individuals who had assessment at the baseline and at the 48-month visits to ensure that we had a sufficient number of individuals to perform analyses by treatment group.

Statistical analyses were performed using Stata14.2 (Stata Inc.). A 2-sided *P* value of less than 0.05 was deemed statistically significant for all analyses.

### Study approval.

The ACCORD study was performed in accordance with the principles of the Declaration of Helsinki. The study proposal was approved by the institutional review boards or ethics committee at the participating centers (see the Supplemental Methods for details), and every participant gave an informed consent prior to participation (31).

## Author contributions

ADK performed the statistical analyses, interpreted the results, participated in the discussion, wrote the first draft of the manuscript, revised the manuscript, and approved the final version of the manuscript. MFY, RSA, and MRR interpreted the results, participated in the discussion, revised the manuscript, and approved the final version of the manuscript. JBET conceived the idea for the study, designed the study, performed the statistical analyses, interpreted the results, participated in the discussion, wrote the first draft of the manuscript, revised the manuscript, and approved the final version of the manuscript. JBET is the guarantor of this work and, as such, had full access to all the data in the study and takes responsibility for the integrity of the data and the accuracy of the data analysis.

## Supplementary Material

Supplemental data

## Figures and Tables

**Table 1:**
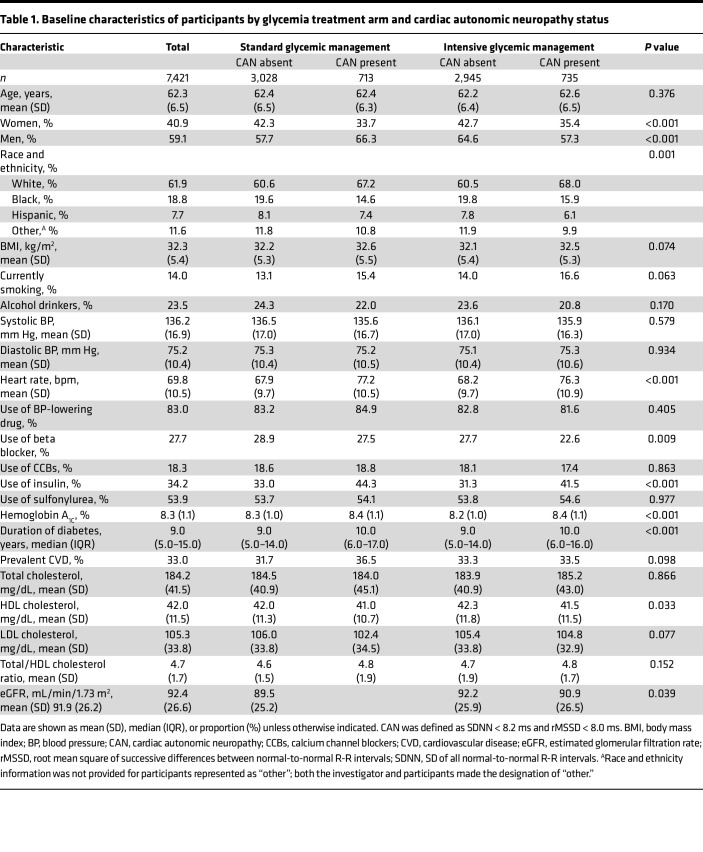
Baseline characteristics of participants by glycemia treatment arm and cardiac autonomic neuropathy status

**Table 2:**
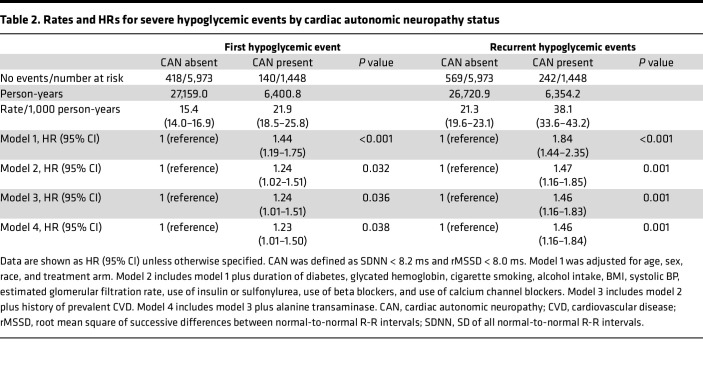
Rates and HRs for severe hypoglycemic events by cardiac autonomic neuropathy status

**Table 3:**
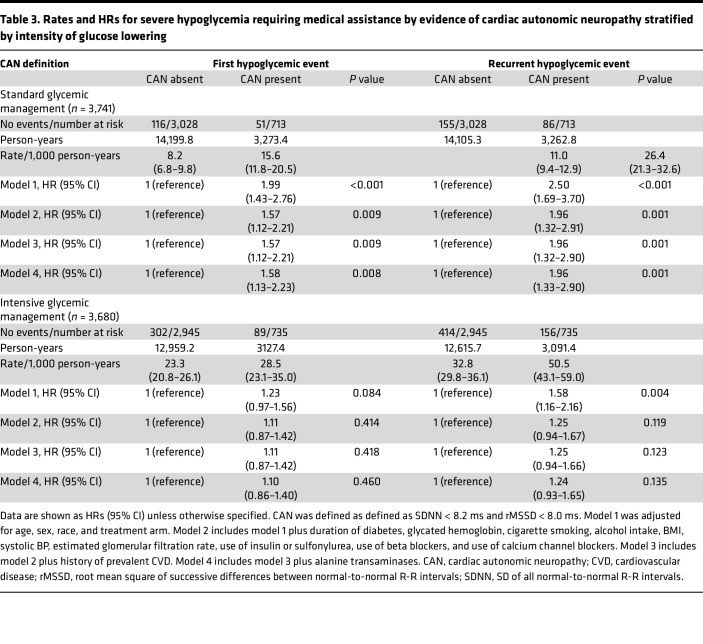
Rates and HRs for severe hypoglycemia requiring medical assistance by evidence of cardiac autonomic neuropathy stratified by intensity of glucose lowering

**Table 4:**
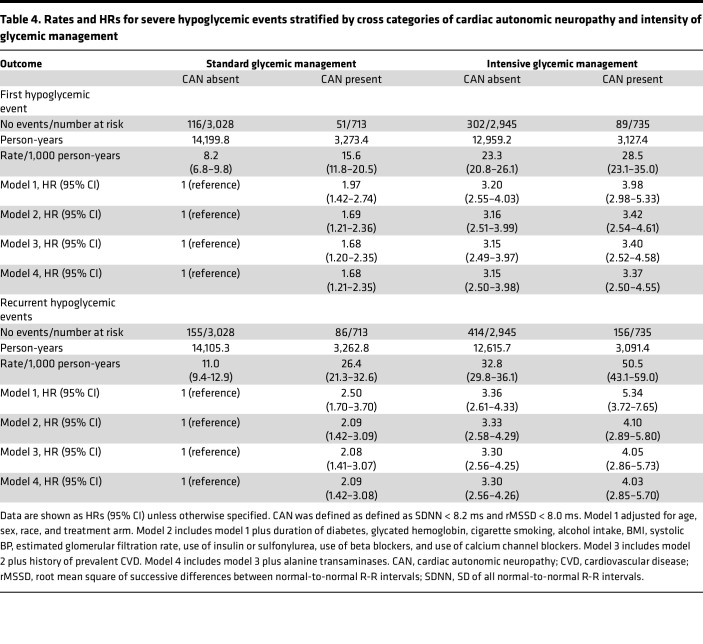
Rates and HRs for severe hypoglycemic events stratified by cross categories of cardiac autonomic neuropathy and intensity of glycemic management

## Version 1 11/01/2022

In-Press Preview

## Version 2 11/22/2022

Electronic publication
