# Supplementary material for: Autonomic dysfunction and risk of severe hypoglycemia among individuals with type 2 diabetes
Source: JCI Insight. 2022 Nov 22;7(22):e156334. doi: 10.1172/jci.insight.156334 (PMC9762508; doi:10.1172/jci.insight.156334)
Supplement: Supplemental data [file jciinsight-7-156334-s006.pdf]

DATA SUPPLEMENT

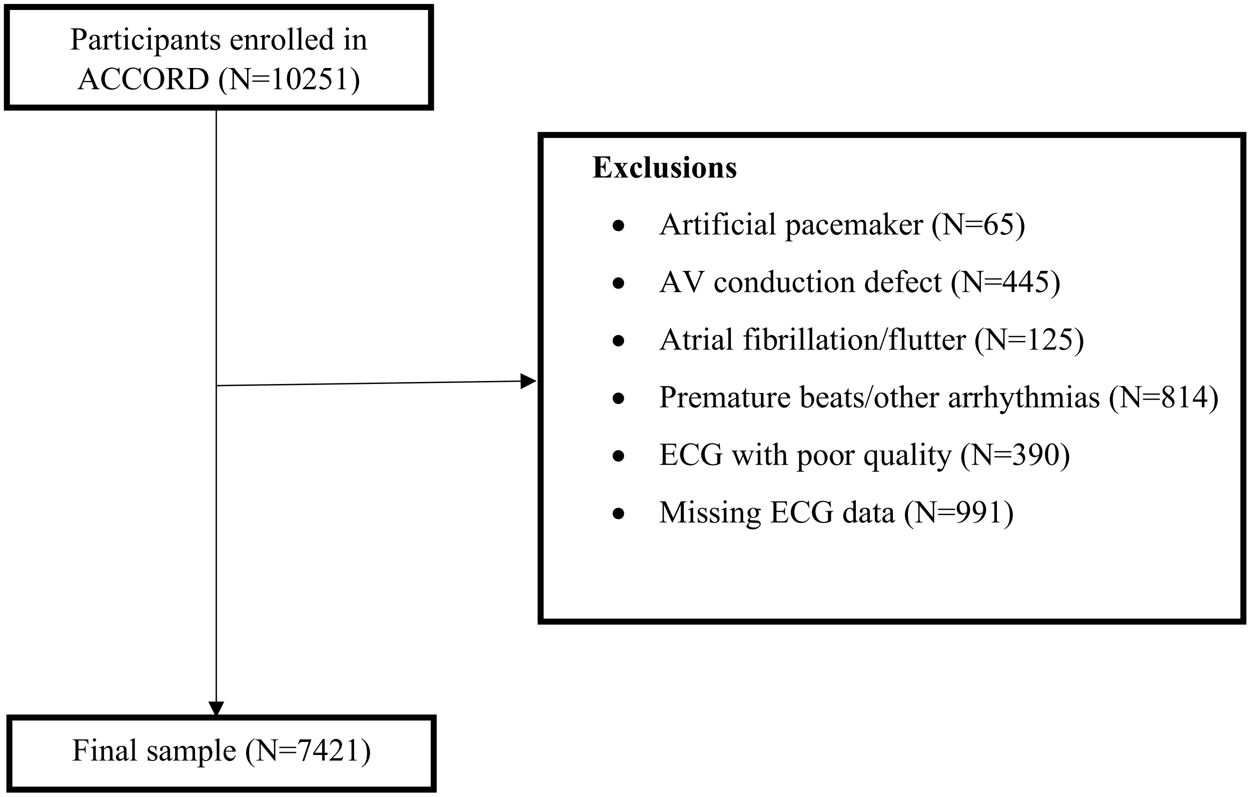

Figure S1. Exclusion criteria for examining the association of cardiac autonomic dysfunction and incidence of severe hypoglycemia among participants enrolled in ACCORD

ACCORD indicates Action to Control Cardiovascular Risk in Diabetes; AV, atrioventricular; ECG, electrocardiogram.

Table S1. Comparison of Participants Excluded to Those Included in the Final Sample

Characteristic	Included	Excluded	*P*-value
N	7421	2830	…
Age, years	62.3 (6.5)	64.0 (7.0)	<0.001
Women, %	40.9	32.3	<0.001
Race/ethnicity, %			0.005
White	61.9	63.5	
Black	18.8	19.7	
Hispanic	7.7	5.8	
Other	11.6	10.9	
Body mass index, kg/m^2	32.3 (5.4)	32.1 (5.5)	0.377
Current smoking, %	14.0	13.8	0.725
Alcohol drinking, %	23.5	25.0	0.113
Systolic BP, mm Hg	136.2 (16.9)	136.8 (17.7)	0.146
Diastolic BP, mm Hg	75.2 (10.4)	74.0 (11.2)	<0.001
Heart rate, bpm	69.8 (10.5)	68.8 (13.4)	<0.001
Use of BP-lowering drug, %	83.0	85.1	0.012
Use of beta blocker, %	27.7	36.6	<0.001
Use of CCB, %	18.3	21.4	<0.001
Use of insulin, %	34.2	36.8	0.016
Use of sulfonylurea, %	53.9	52.2	0.134
Hemoglobin A_{1C}, %	8.3 (1.1)	8.3 (1.1)	0.403
Duration of diabetes, years	9.0 (5.0-15.0)	10.0 (5.0-16.0)	0.001
Prevalent CVD	33.0	41.0	<0.001
Total cholesterol, mg/dL	184.2 (41.5)	180.9 (42.7)	<0.001
HDL-cholesterol, mg/dL	42.0 (11.5)	41.6 (12.0)	0.142
LDL-cholesterol, mg/dL	105.3 (33.8)	103.9 (34.2)	0.055
Total/HDL-cholesterol Ratio	4.7 (1.7)	4.6 (1.6)	0.333
eGFR, mL/min/1.73m^2	91.9 (26.2)	88.8 (29.5)	<0.001

Data are mean (standard deviation), median (interquartile range), or proportion (%) unless otherwise indicated. CAN was defined as SDNN < 8.2 ms and rMSSD < 8.0 ms. BP indicates blood pressure; CAN, cardiac autonomic neuropathy; CCBs, calcium channel blockers; CVD, cardiovascular disease; eGFR, estimated glomerular filtration rate; HDL, high-density lipoprotein; LDL, low-density lipoprotein; rMSSD, root mean square of successive differences between normal-to-normal R-R intervals; SDNN, standard deviation of all normal-to-normal R-R intervals.

Table S2. Baseline Characteristics of Participants Cardiac Autonomic Neuropathy status

Characteristic	Total	CAN Absent	CAN Present	*P*-value
N	7421	5973	1448	…
Age, years	62.3 (6.5)	62.3 (6.5)	62.5 (6.4)	0.227
Women, %	40.9	42.5	34.5	<0.001
Race/ethnicity, %				<0.001
White	61.9	60.5	67.6	
Black	18.8	19.7	15.3	
Hispanic	7.7	7.9	6.8	
Other	11.6	11.8	10.4	
Body mass index, kg/m^2	32.3 (5.4)	32.2 (5.4)	32.6 (5.4)	0.010
Current smoking, %	14.0	13.5	16.0	0.014
Alcohol drinking, %	23.5	24.0	21.4	0.038
Systolic BP, mm Hg	136.2 (16.9)	136.3 (17.0)	135.8 (16.5)	0.286
Diastolic BP, mm Hg	75.2 (10.4)	75.2 (10.4)	75.2 (10.5)	0.978
Heart rate, bpm	69.8 (10.5)	68.1 (9.7)	76.8 (10.7)	<0.001
Use of BP-lowering drug, %	83.0	83.0	83.2	0.824
Use of beta blocker, %	27.7	28.3	25.0	0.013
Use of CCB, %	18.3	18.3	18.1	0.845
Use of insulin, %	34.2	32.1	42.9	<0.001
Use of sulfonylurea, %	53.9	53.7	54.4	0.677
Hemoglobin A_{1C}, %	8.3 (1.1)	8.3 (1.0)	8.4 (1.1)	<0.001
Duration of diabetes, years	9.0 (5.0-15.0)	9.0 (5.0-14.0)	10.0 (6.0-7.0)	<0.001
Prevalent CVD	33.0	32.5	34.9	0.080
Total cholesterol, mg/dL	184.2 (41.5)	184.2 (40.9)	184.6 (44.0)	0.721
HDL-cholesterol, mg/dL	42.0 (11.5)	42.2 (11.6)	41.2 (11.1)	0.007
LDL-cholesterol, mg/dL	105.3 (33.8)	105.7 (33.8)	103.6 (33.7)	0.035
Total/HDL-cholesterol Ratio	4.7 (1.7)	4.6 (1.7)	4.8 (1.8)	0.023
eGFR, mL/min/1.73m^2	91.9 (26.2)	92.3 (26.2)	90.2 (25.9)	0.007

Data are mean (standard deviation), median (interquartile range), or proportion (%) unless otherwise indicated. CAN was defined as SDNN < 8.2 ms and rMSSD < 8.0 ms. BP indicates blood pressure; CAN, cardiac autonomic neuropathy; CCBs, calcium channel blockers; CVD, cardiovascular disease; eGFR, estimated glomerular filtration rate; HDL, high-density lipoprotein; LDL, low-density lipoprotein; rMSSD, root mean square of successive differences between normal-to-normal R-R intervals; SDNN, standard deviation of all normal-to-normal R-R intervals.

Table S3. Rates and Hazard Ratios for Severe Hypoglycemic Events Requiring Any assistance by Cardiac Autonomic Neuropathy status

	First Hypoglycemic Event			Recurrent Hypoglycemic Events		
	**CAN Absent**	**CAN Present**	***P value***	**CAN Absent**	**CAN Present**	***P value***
No Events/No at risk	652/5973	202/1448	…	1056/5973	433/1448	…
Person-years	26401.4	6188.3	…	26720.9	6354.2	…
Rate/1000 person-years	24.7 (22.9-26.7)	32.6 (28.4-37.5)	…	39.5 (37.2-42.0)	68.1 (62.0-74.9)	…
Hazard ratio (95% CI)						
Model 1	1 (Reference)	1.33 (1.13-1.55)	<0.001	1 (Reference)	1.77 (1.36-2.29)	<0.001
Model 2	1 (Reference)	1.14 (0.97-1.34)	0.110	1 (Reference)	1.43 (1.12-1.82)	0.004
Model 3	1 (Reference)	1.14 (0.97-1.34)	0.117	1 (Reference)	1.42 (1.12-1.80)	0.004
Model 4	1 (Reference)	1.14 (0.97-1.34)	0.120	1 (Reference)	1.42 (1.12-1.80)	0.004

Data are hazard ratios (95% CI) unless otherwise specified. CAN was defined as defined as SDNN < 8.2 ms and rMSSD < 8.0 ms. Model 1 adjusted for age, sex, race, and treatment arm; model 2 includes model 1 plus duration of diabetes, glycated hemoglobin, cigarette smoking, alcohol intake, body mass index, systolic blood pressure, estimated glomerular filtration rate, use of insulin or sulfonylurea, use of beta blockers, and use of calcium channel blockers; model 3 includes model 2 plus history of prevalent CVD; model 4 includes model 3 plus alanine transaminases (ALT). CAN. CAN indicates cardiac autonomic neuropathy; CVD, cardiovascular disease; rMSSD, root mean square of successive differences between normal-to-normal R-R intervals; SDNN, standard deviation of all normal-to-normal R-R intervals.

Table S4. Rates and Hazard Ratios for Severe Hypoglycemia Requiring Any assistance by Evidence of Cardiac Autonomic Neuropathy Stratified by Intensity of Glucose Lowering

CAN Definition	First hypoglycemic event			Recurrent hypoglycemic event		
	CAN Absent	CAN Present	*P value*	CAN Absent	CAN Present	*P value*
**Standard Glycemic Management (N=3741)**						
No Events/No at risk	172/3028	69/713		260/3028	129/713	
Person-years	14027.0	3207.9		14105.3	3262.8	
Rate/1000 person-years	12.3 (10.6-14.2)	21.5 (17.0-27.2)		18.4 (16.3-20.8)	39.5 (33.3-47.0)	
Hazard ratio (95% CI)						
Model 1	1 (Reference)	1.78 (1.34-2.37)	<0.001	1 (Reference)	2.21 (1.56-3.13)	<0.001
Model 2	1 (Reference)	1.42 (1.06-1.91)	0.020	1 (Reference)	1.76 (1.24-2.50)	0.002
Model 3	1 (Reference)	1.42 (1.06-1.90)	0.020	1 (Reference)	1.75 (1.24-2.48)	0.002
Model 4	1 (Reference)	1.43 (1.07-1.92)	0.016	1 (Reference)	1.77 (1.25-2.50)	0.001
**Intensive Glycemic Management (N=3680)**						
No Events/No at risk	480/2945	133/735		796/2945	304/735	
Person-years	12374.5	2980.4		12615.7	3091.4	
Rate/1000 person-years	38.8 (35.5-42.4)	44.6 (37.7-52.9)		63.1 (58.9-67.6)	98.3 (87.9-110.0)	
Hazard ratio (95% CI)						
Model 1	1 (Reference)	1.16 (0.96-1.41)	0.126	1 (Reference)	1.61 (1.15-2.26)	0.006
Model 2	1 (Reference)	1.04 (0.85-1.26)	0.733	1 (Reference)	1.31 (0.96-1.78)	0.084
Model 3	1 (Reference)	1.04 (0.85-1.26)	0.735	1 (Reference)	1.31 (0.97-1.77)	0.083
Model 4	1 (Reference)	1.03 (0.84-1.25)	0.788	1 (Reference)	1.30 (0.95-1.77)	0.099

Data are hazard ratios (95% CI) unless otherwise specified. CAN was defined as defined as SDNN < 8.2 ms and rMSSD < 8.0 ms. Model 1 adjusted for age, sex, race, and treatment arm; model 2 includes model 1 plus duration of diabetes, glycated hemoglobin, cigarette smoking, alcohol intake, body mass index, systolic blood pressure, estimated glomerular filtration rate, use of insulin or sulfonylurea, use of beta blockers, and use of calcium channel blockers; model 3 includes model 2 plus history of prevalent CVD. CAN indicates cardiac autonomic neuropathy; CVD, cardiovascular disease; rMSSD, root mean square of successive differences between normal-to-normal R-R intervals; SDNN, standard deviation of all normal-to-normal R-R intervals

Table S5. Rates and Hazard Ratios for Severe Hypoglycemic Events Requiring Any assistance by Cross-Categories of Cardiac Autonomic Neuropathy Status and Glycemic Treatment Arm

Outcome	Standard Glycemic management		Intensive Glycemic management	
	CAN Absent	CAN Present	CAN Absent	CAN Present
**First Hypoglycemic Event**				
No Events/No at risk	172/3028	69/713	480/2945	133/735
Person-years	14027.0	3207.9	12374.5	2980.4
Rate/1000 person-years	12.3 (10.6-14.2)	21.5 (17.0-27.2)	38.8 (35.5-42.4)	44.6 (37.7-52.9)
Hazard ratio (95% CI)				
Model 1	1 (Reference)	1.79 (1.35-2.37)	3.49 (2.90-4.21)	4.07 (3.21-5.16)
Model 2	1 (Reference)	1.54 (1.16-2.04)	3.51 (2.91-4.24)	3.56 (2.79-4.54)
Model 3	1 (Reference)	1.54 (1.16-2.04)	3.50 (2.90-4.23)	3.54 (2.78-4.52)
Model 4	1 (Reference)	1.54 (1.16-2.04)	3.50 (2.90-4.23)	3.52 (2.76-4.49)
**Recurrent Hypoglycemic Events**				
No Events/No at risk	260/3028	129/713	796/2945	304/735
Person-years	14105.3	3262.8	12615.7	3091.4
Rate/1000 person-years	18.4 (16.3-20.8)	39.5 (33.3-47.0)	63.1 (58.9-67.6)	98.3 (87.9-110.0)
Hazard ratio (95% CI)				
Model 1	1 (Reference)	2.23 (1.58-3.15)	3.96 (3.12-5.02)	6.37 (4.34-9.35)
Model 2	1 (Reference)	1.83 (1.30-2.59)	3.92 (3.08-4.98)	5.08 (3.55-7.27)
Model 3	1 (Reference)	1.83 (1.29-2.58)	3.88 (3.06-4.93)	5.01 (3.52-7.14)
Model 4	1 (Reference)	1.83 (1.30-2.59)	3.89 (3.06-4.94)	4.98 (3.48-7.13)

Data are hazard ratios (95% CI) unless otherwise specified. CAN was defined as defined as SDNN < 8.2 ms and rMSSD < 8.0 ms. Model 1 adjusted for age, sex, race, and treatment arm; model 2 includes model 1 plus duration of diabetes, glycated hemoglobin, cigarette smoking, alcohol intake, body mass index, systolic blood pressure, estimated glomerular filtration rate, use of insulin or sulfonylurea, use of beta blockers, and use of calcium channel blockers; model 3 includes model 2 plus history of prevalent CVD; model 4 includes model 3 plus alanine transaminases (ALT). CAN indicates cardiac autonomic neuropathy; CVD, cardiovascular disease; rMSSD, root mean square of successive differences between normal-to-normal R-R intervals; SDNN, standard deviation of all normal-to-normal R-R intervals.

Table S6. Rates and Hazard Ratios for first hypoglycemic event by the use of beta-blockers status

CAN Definition	Not on beta blocker			On beta blocker		
	CAN Absent	CAN Present	*P value*	CAN Absent	CAN Present	*P value*
**Standard Glycemic Management**						
No Events/No at risk	78/ 2,156	30/ 517		38/ 872	21/ 196	
Person-years	10230.9	2398.9		3968.9	874.4	
Rate/1000 person-years	7.6 (6.1-9.5)	12.5 (8.7-17.9)		9.6 (7.0-13.2)	24.0 (15.7-36.8)	
Hazard ratio (95% CI)						
Model 1	1 (Reference)	1.68 (1.10-2.57)	0.016	1 (Reference)	2.79 (1.62-4.81)	<0.001
Model 2	1 (Reference)	1.31 (0.85-2.03)	0.222	1 (Reference)	2.23 (1.29-3.86)	0.004
Model 3	1 (Reference)	1.31 (0.85-2.03)	0.220	1 (Reference)	2.23 (1.28-3.86)	0.004
Model 4	1 (Reference)	1.31 (0.85-2.03)	0.225	1 (Reference)	2.35 (1.35-4.09)	0.002
**Intensive Glycemic Management**						
No Events/No at risk	200/ 2,130	68/ 569		102/ 815	21/ 166	
Person-years	9474.3	2433.1		3484.9	694.3	
Rate/1000 person-years	21.1 (18.4-24.2)	27.9 (22.0-35.4)		29.3 (24.1-35.5)	30.2 (19.7-46.4)	
Hazard ratio (95% CI)						
Model 1	1 (Reference)	1.31 (0.99-1.73)	0.057	1 (Reference)	1.05 (0.65-1.69)	0.842
Model 2	1 (Reference)	1.11 (0.84-1.49)	0.459	1 (Reference)	0.99 (0.61-1.60)	0.959
Model 3	1 (Reference)	1.12 (0.84-1.49)	0.459	1 (Reference)	0.99 (0.61-1.61)	0.973
Model 4	1 (Reference)	1.11 (0.83-1.48)	0.472	1 (Reference)	0.98 (0.61-1.59)	0.946

Data are hazard ratios (95% CI) unless otherwise specified. CAN was defined as defined as SDNN < 8.2 ms and rMSSD < 8.0 ms. Model 1 adjusted for age, sex, race, and treatment arm; model 2 includes model 1 plus duration of diabetes, glycated hemoglobin, cigarette smoking, alcohol intake, body mass index, systolic blood pressure, estimated glomerular filtration rate, use of insulin or sulfonylurea, use of beta blockers, and use of calcium channel blockers; model 3 includes model 2 plus history of prevalent CVD; model 4 includes model 3 plus alanine transaminases (ALT). CAN indicates cardiac autonomic neuropathy; CVD, cardiovascular disease; rMSSD, root mean square of successive differences between normal-to-normal R-R intervals; SDNN, standard deviation of all normal-to-normal R-R intervals.

Table S7. Rates and Hazard Ratios for recurrent hypoglycemic event by the use of beta-blockers status

CAN Definition	Not on beta blocker			On beta blocker		
	CAN Absent	CAN Present	*P value*	CAN Absent	CAN Present	*P value*
**Standard Glycemic Management**						
No Events/No at risk	106/ 2,156	48/ 517		49/ 872	38/ 196	
Person-years	10176.3	2394.9		3928.9	867.9	
Rate/1000 person-years	10.4 (8.6-12.6)	20.0 (15.1-26.6)		12.5 (9.4-16.5)	43.8 (31.9-60.2)	
Hazard ratio (95% CI)						
Model 1	1 (Reference)	1.94 (1.18-3.20)	0.009	1 (Reference)	4.06 (2.18-7.57)	<0.001
Model 2	1 (Reference)	1.51 (0.91-2.50)	0.110	1 (Reference)	2.88 (1.57-5.25)	0.001
Model 3	1 (Reference)	1.51 (0.91-2.50)	0.110	1 (Reference)	2.87 (1.57-5.25)	0.001
Model 4	1 (Reference)	1.51 (0.91-2.51)	0.111	1 (Reference)	3.01 (1.68-5.41)	<0.001
**Intensive Glycemic Management**						
No Events/No at risk	290/ 2,130	128/ 569		124/ 815	28/ 166	
Person-years	9239.6	2402.3		3376.1	689.1	
Rate/1000 person-years	31.4 (28.0-35.2)	53.3 (44.8-63.4)		36.7 (30.8-43.8)	40.6 (28.1-58.8)	
Hazard ratio (95% CI)						
Model 1	1 (Reference)	1.70 (1.18-2.44)	0.004	1 (Reference)	1.16 (0.69-1.94)	0.573
Model 2	1 (Reference)	1.23 (0.88-1.72)	0.217	1 (Reference)	1.11 (0.66-1.87)	0.696
Model 3	1 (Reference)	1.23 (0.88-1.72)	0.219	1 (Reference)	1.11 (0.66-1.87)	0.684
Model 4	1 (Reference)	1.23 (0.89-1.72)	0.215	1 (Reference)	1.11 (0.66-1.86)	0.695

Data are hazard ratios (95% CI) unless otherwise specified. CAN was defined as defined as SDNN < 8.2 ms and rMSSD < 8.0 ms. Model 1 adjusted for age, sex, race, and treatment arm; model 2 includes model 1 plus duration of diabetes, glycated hemoglobin, cigarette smoking, alcohol intake, body mass index, systolic blood pressure, estimated glomerular filtration rate, use of insulin or sulfonylurea, use of beta blockers, and use of calcium channel blockers; model 3 includes model 2 plus history of prevalent CVD; model 4 includes model 3 plus alanine transaminases (ALT). CAN indicates cardiac autonomic neuropathy; CVD, cardiovascular disease; rMSSD, root mean square of successive differences between normal-to-normal R-R intervals; SDNN, standard deviation of all normal-to-normal R-R intervals

Table S8. Effect of change in cardiac autonomic neuropathy (from baseline to 48-months) on the risk of severe hypoglycemic events. (N = 4,111)

CAN Definition	First hypoglycemic event			Recurrent hypoglycemic event		
	**No CAN**	**Progressed to CAN**	**CAN stable**	**No CAN**	**Progressed to CAN**	**CAN stable**
**Standard Glycemic Management (N= 2,059)**						
Hazard ratio (95% CI)						
Model 1	1 (Reference)	1.22 (0.65-2.29)	1.75 (1.08-2.81)	1 (Reference)	1.15 (0.59-2.22)	2.16 (1.26-3.69)
Model 2	1 (Reference)	1.21 (0.64-2.28)	1.41 (0.87-2.30)	1 (Reference)	1.08 (0.56-2.09)	1.70 (0.98-2.95)
Model 3	1 (Reference)	1.21 (0.64-2.29)	1.41 (0.87-2.30)	1 (Reference)	1.08 (0.56-2.09)	1.70 (0.98-2.96)
Model 3 + ALT	1 (Reference)	1.21 (0.64-2.28)	1.42 (0.87-2.31)	1 (Reference)	1.08 (0.56-2.07)	1.71 (0.98-2.97)
**Intensive Glycemic Management (N= 2,052)**						
Hazard ratio (95% CI)						
Model 1	1 (Reference)	1.13 (0.75-1.70)	1.15 (0.84-1.59)	1 (Reference)	1.06 (0.65-1.71)	1.37 (0.89-2.11)
Model 2	1 (Reference)	1.10 (0.72-1.67)	1.06 (0.76-1.48)	1 (Reference)	1.01 (0.62-1.64)	1.05 (0.72-1.55)
Model 3	1 (Reference)	1.10 (0.72-1.67)	1.05 (0.76-1.47)	1 (Reference)	1.01 (0.62-1.64)	1.05 (0.71-1.54)
Model 3 + ALT	1 (Reference)	1.07 (0.71-1.63)	1.02 (0.73-1.42)	1 (Reference)	0.99 (0.61-1.60)	1.01 (0.69-1.49)

Data are hazard ratios (95% CI) unless otherwise specified. CAN was defined as defined as SDNN < 8.2 ms and rMSSD < 8.0 ms. Model 1 adjusted for age, sex, race, and treatment arm; model 2 includes model 1 plus duration of diabetes, glycated hemoglobin, cigarette smoking, alcohol intake, body mass index, systolic blood pressure, estimated glomerular filtration rate, use of insulin or sulfonylurea, use of beta blockers, and use of calcium channel blockers; model 3 includes model 2 plus history of prevalent CVD. CAN indicates cardiac autonomic neuropathy; CVD, cardiovascular disease; rMSSD, root mean square of successive differences between normal-to-normal R-R intervals; SDNN, standard deviation of all normal-to-normal R-R intervals

Clinical center networks (CCNs) and clinical sites:
Canadian CCN: Population Health Research Institute, Hamilton General Hospital, Canadian Diabetes Outcome Researchers (CANDOR Network), Hamilton, Ontario, Canada.
Canadian clinical sites
McMaster Medical Centre, Hamilton, Ontario,
Canada,Six Nations Health Services, Ohsweken, Ontario, Canada
Diabetes, Hypertension and Cholesterol Centre, University of Calgary, Calgary, Alberta, Canada, Memorial University of Newfoundland, St. John's, Newfoundland, Canada, University of Alberta, Edmonton, Alberta, Canada,
Centre de Recherche Clinique de Laval, Laval, Quebec, Canada
St. Joseph's Health Care London, London, Ontario, Canada
Ottawa Hospital, Division of Endocrinology and Metabolism, Ottawa, Ontario, Canada
Royal Victoria Hospital, Montreal, Quebec, Canada
St. Michael's Hospital Health Centre, Toronto, Ontario, Canada
Vancouver General Hospital, Vancouver, British Columbia, Canada
Diabetes Research Group, Winnipeg, Manitoba, Canada
Queen Elizabeth II Health Sciences Centre, Halifax, Nova Scotia, Canada

Western CCN: University of Washington, Seattle, WA:
Western clinical sites:
Northridge Hospital Medical Center, Cardiovascular Center, Northridge, CA
White Memorial Medical Center, Clinical Hypertension Services, Los Angeles, CA
University of Washington Medical Center at Roosevelt, Family Medical Center, Seattle, WA
Idaho State University, Department of Family Medicine, Pocatello, ID
Naval Medical Center San Diego, Cardiology Division, San Diego, CA
Oregon Health & Science University, Section of Diabetes, Portland, OR
Washington State University, Spokane, WA
Kaiser Endocrine Clinic, San Diego, CA
Whittier Institute for Diabetes, Clinical Trials Department, La Jolla, CA

Minnesota-Iowa CCN: Berman Center for Outcomes & Clinical Research, Minneapolis, MN
Minnesota-Iowa clinical sites:
Hennepin ACCORD Clinic, Minneapolis, MN
International Diabetes Center at Park Nicollet, St. Louis Park, MN
Ellie Strock. University of Minnesota, Minneapolis, MN
University of Minnesota, Phalen Village Clinic, St. Paul, MN
Riverside Health Partners Clinic, Department of Endocrinology, Minneapolis, MN
University of Iowa, Health Care Diabetes Clinical Research and Programs, Iowa City, IA

Ohio-Michigan CCN: Case Western Reserve University, Division of Clinical and Molecular Endocrinology, Cleveland, OH
Ohio-Michigan clinical sites:
University Hospitals of Cleveland, Division of Endocrinology, and University Hospitals Weslake Medical, Cleveland, OH
St. Vincent Charity Hospital, Lipid Research Center, Cleveland, OH
University Suburban Health Center, South Euclid, OH

Cleveland Veterans Affairs (VA) Medical Center (VAMC), Department of Medicine, and Ravenna Community Based Outpatient Clinic, Cleveland,
The Cleveland Clinic Foundation and Lakewood Hospital Professional Building, Cleveland, OH
Your Diabetes Endocrine Nutrition Group, Mentor, OH
Medical University of Ohio, Department of Medicine, Ruppert Health Center, Toledo, OH
The Ohio State University Medical Center, Division of Endocrinology, Diabetes and Metabolism, Columbus, OH
University of Cincinnati/VA Medical Center, Research Service, Cincinnati, OH
Henry Ford Health System–New Center One, Detroit, MI
Grunberger Diabetes Institute, Bloomfield Hills, M

Northeastern CCN: Columbia University College of Physicians and Surgeons, New York, NY
Northeastern clinical sites:
Jacobi Medical Center, Bronx, NY
Albert Einstein General Clinical Research Center, Bronx, NY
Cornell Internal Medicine Associates, New York, NY
The Diabetes Care and Information Center of New York, Flushing, NY
The Cooper Health System, Pennsville, NJ
Great Lakes Medical Clinic Research, Westfield, NY
Ambulatory Care Network at Columbia University, New York, NY
Irving Diabetes Research Unit, New York, NY
State University of New York Downstate Medical Center, Brooklyn, NY
Kings County, Brooklyn, NY
Cooper Clinical Trials Center, The Cooper Health System, Camden, NJ

Southeastern CCN: Wake Forest University School of Medicine, Department of Public Health Sciences, Winston-Salem
Southeastern clinical sites:
Duke University Medical Center, Durham, NC
Constant Care, Inc., Valdosta, GA
Wake Forest University School of Medicine, Department of Geriatrics/Gerontology, Winston-Salem, NC
Downtown Health Plaza, Winston-Salem, NC
University of North Carolina, Diabetes Care Center, Chapel Hill, NC
Holston Medical Group, Kingsport, TN
Carolinas Medical Center Family Practice, Charlotte, NC
Robeson Health Care Corporation, Fairmont Clinic, Fairmont, NC
Wake Forest University School of Medicine, Departments of Internal Medicine and Endocrinology, Winston-Salem, NC
Tulane University Health Science Center, New Orleans, LA
Kaiser Permanente, Clinic Atlanta Crescent Medical Center, Tucker, GA

VA CCN: Memphis VAMC, Memphis, TN
VA clinical sites:
Memphis VAMC, Hypertension/Lipid Research Clinic, Memphis, TN
Atlanta VAMC Medical Service, Decatur, GA

Johnson VAMC, Primary Care, Charleston, SC
Rehman. G. V. (Sonny) Montgomery VAMC, Research Department, Jackson, MS
VA NY Harbor Healthcare System, New York, NY
Washington VAMC, Washington, DC
St. Louis VAMC, St. Louis, MO
Central Arkansas Clinic Healthcare System, Little Rock, AR
